# Activated Circulating T Follicular Helper Cells Are Associated with Disease Severity in Patients with Psoriasis

**DOI:** 10.1155/2016/7346030

**Published:** 2016-09-28

**Authors:** Ying Wang, Lili Wang, Haoyu Yang, Weichang Yuan, Jingyi Ren, Yanping Bai

**Affiliations:** ^1^Clinical Institute of China-Japan Friendship Hospital, Graduate School of Peking Union Medical College, No. 2, Yinghua East Street, Chaoyang District, Beijing 100029, China; ^2^Department of Dermatology & Venerology, China-Japan Friendship Hospital, No. 2, Yinghua East Street, Chaoyang District, Beijing 100029, China; ^3^Beijing University of Chinese Medicine, No. 11, Bei San Huan East Road, Chaoyang District, Beijing 100029, China; ^4^Department of Orthopedics, Taian Chinese Medicine Hospital, No. 58, Dongyue Street, Taishan District, Taian 271000, China

## Abstract

Circulating T follicular helper (cTfh) cells are known to be involved in numerous immune-mediated diseases, but their pathological role in psoriasis is less fully investigated. Herein, we aimed to identify whether cTfh cells contributed to the pathogenesis of psoriasis. The frequency and function of cTfh cells were compared between patients with psoriasis vulgaris and healthy controls, and the infiltration of Tfh cells was detected between lesional and nonlesional skin tissues of psoriasis patients. Moreover, the dynamic change of cTfh cells before and after acitretin treatment was evaluated. Our results showed both increased frequency and activation (indicated by higher expression of ICOS, PD-1, HLA-DR, and Ki-67 and increased production of IL-21, IL-17, and IFN-*γ*) of cTfh cells in psoriasis patients. Compared with nonlesional skin tissues of psoriasis patients, the number of infiltrated Tfh cells was significantly increased in psoriasis lesions. In addition, positive correlations between the percentage of cTfh, functional markers on cTfh cells in peripheral blood and disease severity were noted. Furthermore, the frequency of cTfh cells and the levels of cytokines secreted by cTfh cells were all significantly decreased after 1-month treatment.

## 1. Introduction

Despite years of efforts to develop new agents, the recurrence rate and adverse effects related to long-term agent use remain significant in patients with psoriasis [1]. After decades' study, it is generally accepted that psoriasis is a T cell-mediated disease [2]. In addition, though it was previously assumed that Th1 and Th2 cells were the major cells contributing to the development of psoriasis, increasing evidence in recent years showed that Th17 cells played a vital role in the pathogenesis of psoriasis [3]. Clinical trials have reported that the biological agents targeting IL-17, such as secukinumab, brodalumab, and ixekizumab, are effective and safe in patients with psoriasis vulgaris [4]. Except for Th17, Th9 and Th22 cells, which have a close interaction with Th17 [5, 6], were also found increased in psoriasis lesions [7, 8]. Therefore, these newly discovered T cell subsets and their interactions possibly provide a new insight into the molecular mechanism of psoriasis.

T follicular helper (Tfh) cells, which are located in the germinal centers (GCs), are another specialized subset of CD4^+^ T cells. High expression levels of chemokine CXC receptor 5 (CXCR5), inducible T cell costimulator (ICOS), programmed cell death protein-1 (PD-1), and the transcription factors B cell lymphoma 6 (Bcl-6) are distinctive characteristics of Tfh cells [9]. The function of Tfh cells has been found to be closely related to several factors, such as the expression of ICOS and PD-1 and the secretion of IL-21 [10, 11]. Recently, circulating T follicular help (cTfh) cells have been found in human blood, which are considered counterparts to Tfh cells in GCs. cTfh cells share similar markers and have familiar function with Tfh cells in GCs [12]. Many reports have shown that the dysregulated behavior of cTfh cells contributes to numerous autoimmune diseases and infectious diseases, such as systemic lupus erythematosus (SLE) [13], rheumatoid arthritis [14], pulmonary tuberculosis [15], and human immunodeficiency virus (HIV) infection [16]. However, the role of cTfh cells in psoriasis remains lacking, and it is still unclear whether the function of cTfh cells is changed in psoriasis.

To address these issues, we comprehensively explored the frequency, phenotype, and function of CXCR5^+^CD4^+^ cTfh cells in patients with psoriasis vulgaris (PV). In addition, we also investigated the infiltration of Tfh cells in lesional and nonlesional skin tissues of psoriasis patients. To further understand the role of cTfh cells in the pathogenesis of psoriasis, we analyzed the frequency and function of cTfh cells in patients with psoriasis vulgaris before and after 1-month treatment.

## 2. Materials and Methods

### 2.1. Study Subjects

Blood samples were obtained from 32 patients with psoriasis vulgaris and 13 healthy donors. The diagnosis of psoriasis vulgaris was based on the established criteria of Nestle et al. [17]. Psoriasis area severity index (PASI) score was used to assess the disease activity in psoriasis [18]. Coexisting other autoimmune diseases, systemic diseases, or active infections were excluded in all of these subjects. In addition, no patients had received any systemic therapy for at least one month before enrolment. After enrolment, 24 of the 32 patients with moderate to severe psoriasis received 20 mg of oral acitretin (Fangxi, Chongqing Huapont Pharm. Co., Ltd.) once daily and topical therapy of calcipotriol ointment (Daivonex, LEO Laboratories Limited, Ireland) and mometasone furoate cream (Eloson, Shanghai Xianlingbaoya Pharm. Co., Ltd.) once daily for 4 weeks. Lesional and nonlesional skin tissues were obtained from 10 patients with psoriasis vulgaris. Normal skin tissues were collected from 5 healthy individuals. The study protocol was approved by China-Japan Friendship Hospital Research Ethics Committee, and written informed consent was obtained from each participant. The clinical background of the patients was shown in Table 1.

### 2.2. Fluorescence Antibodies and Flow Cytometry

The frequencies of CXCR5^+^CD4^+^ cTfh cells, as well as the functional molecules of cTfh cells, in 32 psoriasis patients and 13 healthy controls were detected by flow cytometry. Peridinin chlorophyll protein- (PerCP-) conjugated anti-CD4 and anti-CD8, fluorescein isothiocyanate- (FITC-) conjugated anti-CXCR5, phycoerythrin- (PE-) conjugated anti-ICOS and anti-HLA-DR, and allophycocyanin- (APC-) conjugated anti-IL-10, anti-IFN-*γ*, and Ki-67 were purchased from BD Bioscience and BD Pharmingen (San Diego, CA, USA). APC-conjugated anti-PD-1, APC/cy7-conjugated anti-CD3, and PE-conjugated anti-IL-21 were purchased from Biolegend (San Diego, CA, USA). PE-conjugated anti-IL-17A was purchased from eBioscience (San Diego, CA, USA). To explore the frequency and function of cTfh cells, one sample of peripheral blood (200 *μ*L) was incubated for 30 min with anti-CD4-PerCP, anti-CXCR5-FITC, anti-PD-1-APC, and anti-ICOS-PE. In addition, anti-CD4-PerCP, anti-CXCR5-FITC, anti-Ki-67-APC, and anti-HLA-DR-PE were added to another sample of freshly heparinized blood sample for 30 min. Then the cells were washed and analyzed by flow cytometry. For intracellular cytokines staining, 300 *μ*L heparinized peripheral blood and 700 *μ*L RPMI1640 medium supplemented with 10% fetal calf serum were incubated for 6 hours with phorbol 12-myristate 13-acetate (PMA, 300 ng/mL, Sigma-Aldrich, St. Louis, MO) and ionomycin (1 *μ*g/mL, Sigma-Aldrich, St. Louis, MO). Then, these cells were stained with anti-CD8-PerCP, anti-CD3-APC/Cy7, and anti-CXCR5-FITC for 20 min at room temperature. Subsequently, these cells were fixed and permeabilized for 50 min at 4°C with eBioscience Fixation/Permeabilization Buffer (eBioscience, San Diego, CA, USA). The samples were then incubated with anti-IL-21-PE and anti-IFN-*γ*-APC or stained with anti-IL-17A-PE and anti-IL-10-APC for 20 min at room temperature. Finally, these cells were fixed in 1% paraformaldehyde and acquired by a multicolor flow cytometry. FlowJo software (Tritar, USA) was used to analyze the data.

### 2.3. Immunohistochemistry

Lesional and nonlesional skin tissues of 10 psoriasis patients and healthy skin tissues from 5 healthy donors were, respectively, collected. These tissues were analyzed by using immunochemical staining with anti-CD4 (Zhongshan Goldenbridge Biotech, Beijing, China) and anti-CXCR5 (Abcam, Cambridge, UK). Paraffin-embedded, formalin-fixed skin tissue was cut into 5 *μ*m sections and placed on polylysine-coated slides. Antigen retrieval was achieved via pressure cooking for 10 min in citrate buffer (pH 6.0). Sections were incubated with anti-CD4 and anti-CXCR5 and then incubated with biotinylated goat-rabbit antibody (Zhongshan Goldenbridge Biotech, Beijing, China). The avidin-biotin-peroxidase system with 3-amino-9-ethylcarbazole (AEC) (brown color) or vector blue (blue color) as substrates was used to perform double staining. CXCR5 and CD4 double positive cells in the skin tissues were identified as Tfh cells. The number of Tfh cells was evaluated quantitatively by 2 independent observers from 3 representative fields (200x). In addition, to further determine Tfh cells in psoriasis lesions, a dual label immunofluorescence technology was performed. The skin tissues were incubated overnight with primary antibodies, including mouse anti-human CD4 (diluted 1 : 10, Abcam; Cambridge, UK) and rabbit anti-human CXCR5 (diluted 1 : 10; Abcam, Cambridge, UK). Then slides were incubated with secondary antibodies (rabbit anti-mouse FITC-conjugated IgG Ab, rhodamine-conjugated goat anti-rabbit IgG Ab, and DAPI for nucleic acid staining) for 45 minutes at room temperature.

### 2.4. Statistical Analysis

Statistical analysis was performed using SPSS 20.0 software (SPSS, Chicago, IL, USA) and the data was presented as the mean values ± standard deviation. The statistical difference between the 2 groups was evaluated with a Mann–Whitney* U* test, whereas the statistical difference between the same individual across patient group was determined by Wilcoxon matched pairs test. Partial correlation was used to analyze the correlation of cTfh frequency and PASI score. Spearman's correlation was used to analyze the association between the other variables. For all tests, *P* < 0.05 was considered to be significant.

## 3. Results

### 3.1. cTfh Cells Are Significantly Increased in Patients with Psoriasis Vulgaris

**Figure 1:**
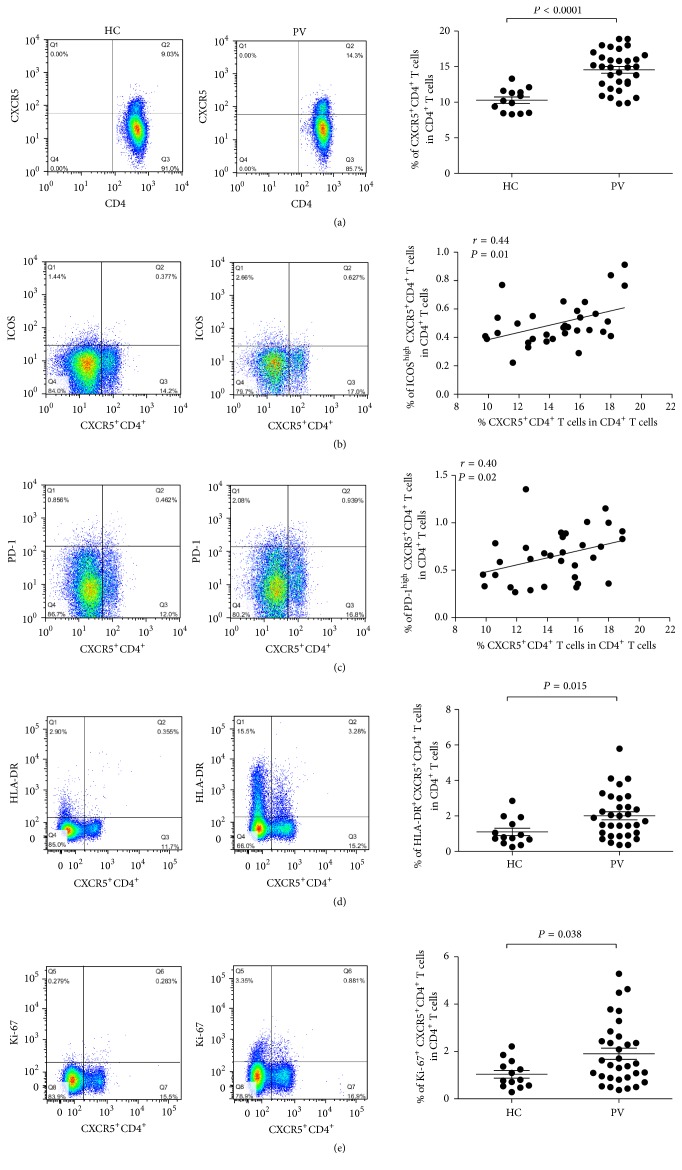
Increased frequency of circulating CXCR5^+^CD4^+^ Tfh (cTfh) cells in patients with psoriasis vulgaris. (a) Comparison of the percentages of cTfh cells in patients with psoriasis vulgaris (PV) and healthy controls (HC). The cTfh cell frequency in patients with psoriasis vulgaris is significantly higher than in healthy controls. (b) ICOS expression on cTfh cells in patients with psoriasis vulgaris and healthy controls. Compared with healthy controls, ICOS expression on cTfh cells is significantly increased and has a positive correlation with the frequency of cTfh cells in psoriasis patients. (c) The levels of PD-1 on cTfh cells in patients with psoriasis vulgaris and healthy controls. The frequency of PD-1^high^CXCR5^+^CD4^+^/CD4^+^ T cells is statistically increased and associated with cTfh cell frequency in psoriasis patients. Each dot represents one participant. *P* values are shown. (d) The expression of HLA-DR on cTfh cells in patients with psoriasis vulgaris and healthy controls. The expression of HLA-DR on cTfh cells was significantly higher in psoriasis patients than in healthy controls. (e) Ki-67 expression on cTfh cells in patients with psoriasis vulgaris and healthy controls. Compared with healthy controls, there was a higher level of Ki-67 expression on cTfh cells in psoriasis patients.

As shown in Figure 1(a), the frequency of cTfh cells was significantly increased in patients with psoriasis vulgaris compared with healthy individuals (14.55 ± 2.67% versus 10.29 ± 1.63%; *P* < 0.0001). In addition, ICOS and PD-1 are two important surface markers on cTfh cells and have critical roles in the differentiation of cTfh cells. Thus, we investigated the expression of these makers in psoriasis. Our data showed that the levels of ICOS and PD-1 expression on cTfh cells were positively correlated with the percentage of cTfh cells (Figure 1(b), *r* = 0.44 and *P* = 0.01; Figure 1(c), *r* = 0.40 and *P* = 0.02, resp.). To further investigate whether cTfh cells were activated in psoriasis, the expression of HLA-DR and Ki-67 on cTfh cells were detected. Our results demonstrated that there were higher levels of HLA-DR and Ki-67 expression on cTfh cells in patients with psoriasis vulgaris (Figure 1(d), 2.01 ± 1.27% versus 1.10 ± 0.76%; *P* = 0.015; Figure 1(e), 1.90 ± 1.34% versus 1.03 ± 0.58%; *P* = 0.038, resp.).

**Figure 2:**
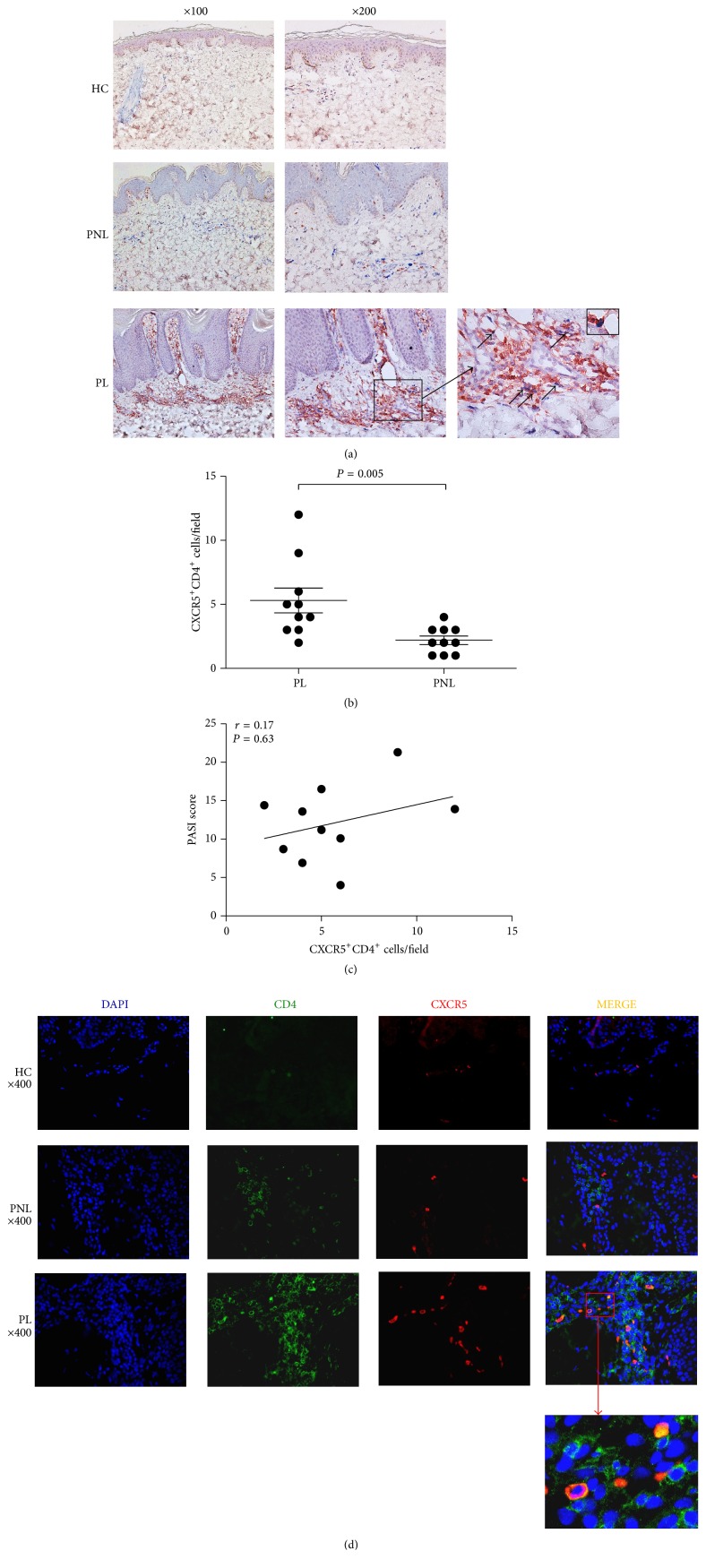
Higher infiltration of Tfh cells in psoriasis lesions. (a) Representative immunohistochemical staining of Tfh in psoriasis lesions (PL), nonlesional skin tissues of psoriasis patients (PNL), and normal skin tissues of healthy controls (HC). Tfh cells are double stained for CD4 (brown, on cell membrane) and CXCR5 (blue, on cell membrane). There are no Tfh cells infiltrated in skin tissues of healthy controls. (b) The number of Tfh cells in psoriasis lesions is significantly increased compared with nonlesional skin tissues of psoriasis patients, which was evaluated quantitatively by 2 independent observers from 3 representative fields (200x). (c) The number of infiltrated Tfh cells in psoriasis lesions is not associated with disease severity, as assessed by psoriasis area severity index (PASI) score. *P* value is shown. (d) Costaining of CD4 and CXCR5 in skin tissues from psoriasis patients and healthy controls by immunofluorescence. Representative staining shows CD4 (green) and CXCR5 (red) cells. DAPI was used to counterstain nuclear DNA (blue). The 2-color merged panels were shown with colocalization visible in yellow. Representative staining at an original magnification of 400x is shown.

Little information is available on the characteristics of Tfh cells infiltrating in psoriasis lesions. Thus, the numbers of Tfh cells in lesional and nonlesional skin tissues of psoriasis patients were first investigated by immunohistochemical double staining in our study. As shown in Figure 2(a), there were no Tfh cells (CD4^+^ and CXCR5^+^ double positive cells) in healthy donor skin tissue. In contrast, we detected an extensive infiltration of Tfh cells in psoriasis lesions. The number of Tfh cells in psoriasis lesions was significantly higher than that in nonlesional skin tissues of psoriasis (Figure 2(b), 5.6 ± 3 versus 2.3 ± 1.2; *P* = 0.005). However, our results demonstrated that although the number of Tfh cells was significantly increased in psoriasis lesions, there was no significant correlation between the number of infiltrating Tfh cells and PASI score in psoriasis (Figure 2(c), *r* = 0.17 and *P* = 0.63). Double-staining immunofluorescence further identified the higher infiltration of CXCR5^+^CD4^+^ T cells in lesions of psoriasis patients (Figure 2(d)).

### 3.2. cTfh Cells Produce Higher Levels of Cytokines in Patients with Psoriasis Vulgaris

**Figure 3:**
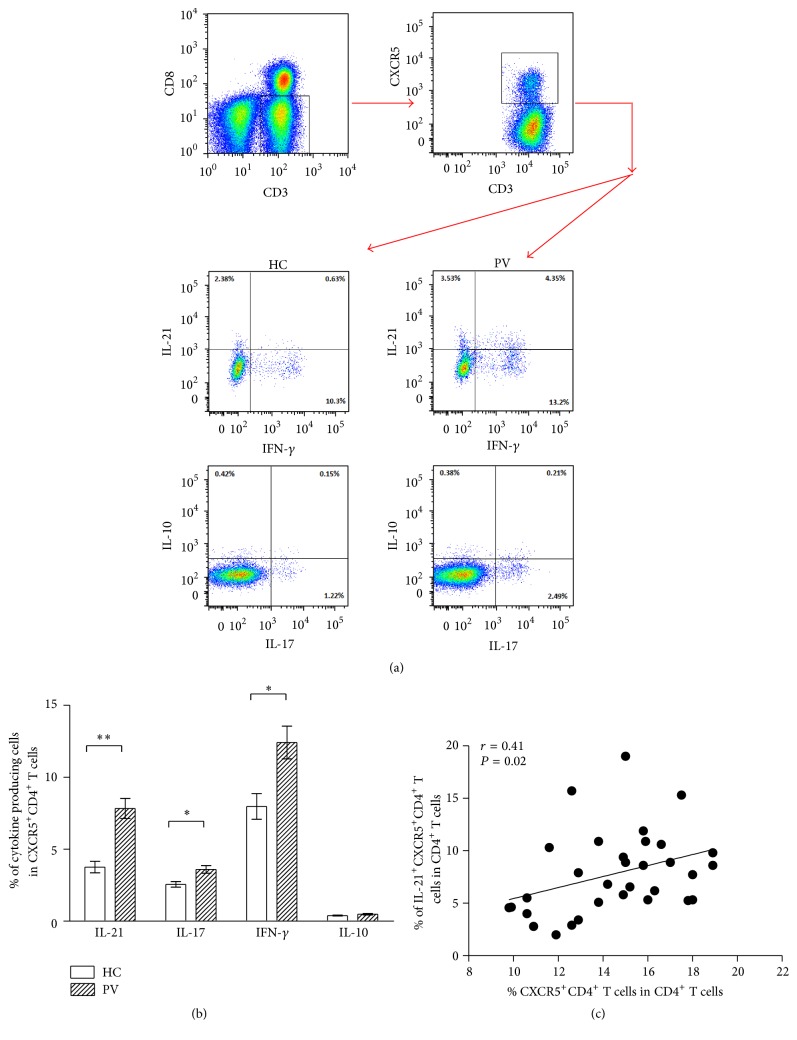
Cytokine production by cTfh cells in patients with psoriasis vulgaris and healthy controls. (a) Represent profiles of IL-21, IFN-*γ*, IL-17, and IL-10 secreted by cTfh cells in psoriasis patients and healthy controls. (b) cTfh cells produce more IL-21, IFN-*γ*, and IL-17 in psoriasis patients than in healthy controls, whereas the secretion of IL-10 shows no significant difference between two groups. ^*∗*^ *P* < 0.05; ^*∗∗*^ *P* < 0.01. (c) The higher expression of IL-21 on cTfh cells is positively correlated with the frequency of cTfh cells in patients with psoriasis vulgaris (*P* < 0.05).

Previous studies have reported that numerous cytokines, especially IL-21, have crucial effects on Tfh cell function. As described above, the frequency of cTfh cells was increased in psoriasis. However, it is unclear whether the function of cTfh cells is changed in patients with psoriasis vulgaris. To answer this question, we detected the levels of cytokines, including IL-21, IFN-*γ*, IL-17, and IL-10, secreted by cTfh cells. In our study, the frequency of IL-21^+^CXCR5^+^CD4^+^ T cells was significantly higher in psoriasis patients than in healthy controls (Figure 3(b), 7.83 ± 3.94% versus 3.76 ± 1.46%; *P* = 0.0003). Additionally, the levels of IL-17 and IFN-*γ* secreted by cTfh cells were also significantly increased in patients with psoriasis vulgaris compared with healthy individuals (Figure 3(b), 3.60 ± 1.54% versus 2.56 ± 0.70%; *P* = 0.025; 12.42 ± 6.45% versus 7.97 ± 3.24%; *P* = 0.033, resp.). However, the secretion of IL-10 by cTfh cells showed no significant difference between psoriasis patients and healthy controls (Figure 3(b), 0.48 ± 0.27% versus 0.38 ± 0.11%; *P* = 0.425). Furthermore, our data represented that the IL-21 production of cTfh cells had positive correlation with the percentage of cTfh cells (Figure 3(c), *r* = 0.41 and *P* = 0.02).

### 3.3. Higher Frequency of cTfh Cells Was Positively Associated with Disease Severity in Psoriasis

**Figure 4:**
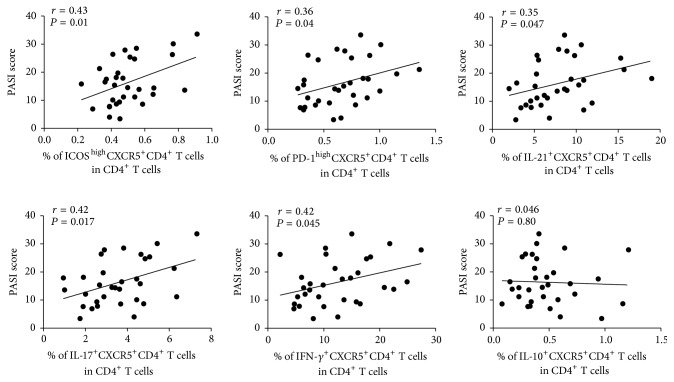
Correlations between the levels of functional markers on cTfh cells and PASI score. The ICOS and PD-1 expression on cTfh cells have positive correlations with PASI score. The positive relationships are also found between the levels of IL-21, IL-17, and IFN-*γ* secreted by cTfh cells and PASI score. However, the IL-10 secreted by cTfh cells has no relationship with PASI score in psoriasis patients. Correlative coefficients and *P* values are shown.

Next, we want to investigate whether cTfh cells associate with disease severity in psoriasis, assessed by PASI score. However, it is generally accepted that aging affects the function of immune system. To address this question, we used partial correlation to analyze the relationship of the frequency of cTfh cells and PASI score in psoriasis. Our results showed that when the age or disease duration was considered as control variables, positive relationship was found between the frequency of cTfh cells and PASI score (Tables 2(a) and 2(b), *P* = 0.014; *P* = 0.029, resp.). Furthermore, we also investigated the correlations between PASI score and the levels of functional markers observed on cTfh cells, including ICOS, PD-1, IL-21, IL-17, IFN-*γ*, and IL-10. Our results demonstrated that these functional markers except IL-10 positively correlated with PASI score in psoriasis (Figure 4, *r* = 0.43 and *P* = 0.01; *r* = 0.36 and *P* = 0.04; *r* = 0.35 and *P* = 0.047; *r* = 0.42 and *P* = 0.017; *r* = 0.42 and *P* = 0.045; *r* = 0.046 and *P* = 0.80, resp.). Thus, our results implied that cTfh cells played an important role in the pathogenesis of psoriasis and may be a biomarker for evaluating the disease severity.

### 3.4. The Frequency of cTfh Cells Was Significantly Decreased after Treatment

**Figure 5:**
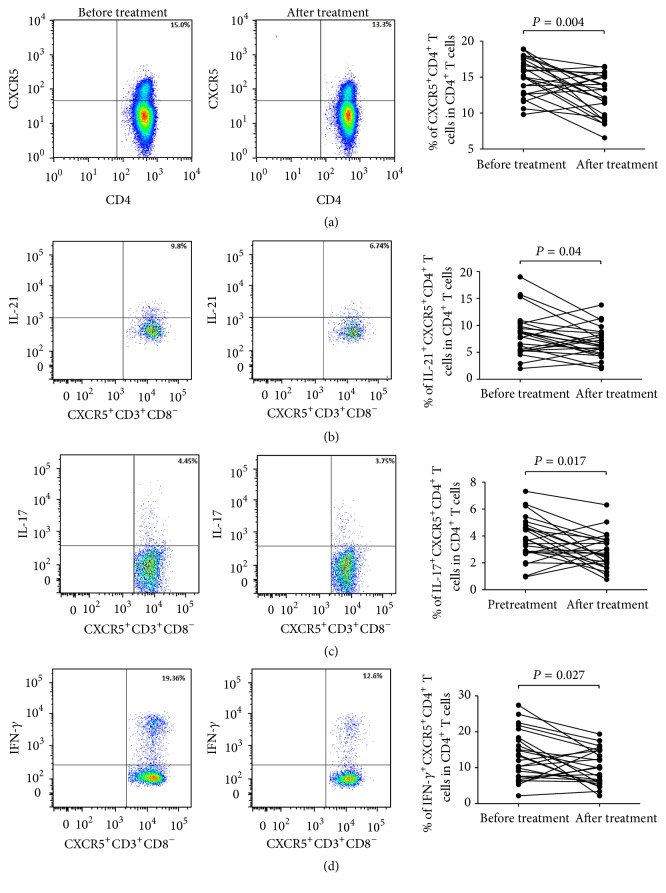
The frequency and cytokine production of cTfh cells before and after treatment. (a) After 1-month treatment, with alleviation of disease severity, the frequency of cTfh cells is significantly decreased in psoriasis patients. (b, c, and d) The levels of IL-21, IL-17, and IFN-*γ* produced by cTfh are all lower than those before treatment.* P* values are shown.

Acitretin is a classic agent for the treatment of psoriasis. Previous studies have shown that acitretin was effective in patients with psoriasis. To further understand the role of cTfh cells in psoriasis, we investigated the function and frequency of cTfh cells in 24 patients with moderate to severe plaque psoriasis before and after 1-month treatment with acitretin and a topical therapy. All subjects did not receive any immunosuppressive agents during follow-up period. Our results demonstrated that the frequency of cTfh cells was significantly decreased in patients after 1-month treatment (Figure 5(a), 15.02 ± 2.63% versus 12.38 ± 2.88%; *P* = 0.004). In addition, there was also significant decline in the secretion of IL-21, IL-17, and IFN-*γ* by cTfh cells after treatment (Figures 5(b), 5(c), and 5(d), 8.35 ± 4.04% versus 6.73 ± 2.88%; *P* = 0.04; 3.80 ± 1.62% versus 2.84 ± 1.30%; *P* = 0.017; 13.31 ± 6.76% versus 10.08 ± 4.92%; *P* = 0.027, resp.), suggesting that cTfh cells are related to disease severity and may be a potential therapeutic target in psoriasis.

## 4. Discussion

CD4^+^ T cells are critical component of immune system and have been demonstrated to play a central role in the pathogenesis of psoriasis [19]. Naïve CD4^+^ T cells can differentiate into distinct lineages driven by different cytokines in the environments, and these subsets have specialized functions [20]. The newly found subpopulations of CD4^+^ T cells, such as Th17, Th22, and regulatory T cells, have enriched our understanding of the immune state of psoriasis [21, 22]. cTfh cells are recently discovered CD4^+^ T cells subset and have been found to be associated with the development of many diseases [23]. However, the researches on the role of cTfh cells in psoriasis are less. In the present study, we not only investigated the frequency of cTfh cells but also further explored the function of cTfh cells in patients with psoriasis vulgaris. Furthermore, the histological characteristics of Tfh cells in psoriasis lesions were firstly analyzed by our group. Finally, to further confirm the role of cTfh cells in psoriasis in vivo and to shed light on the corresponding therapeutic significance, we also firstly observed the dynamic changes of cTfh cell frequency and function in patients before and after treatment.

In clinical trials, cTfh cells have been shown to be increased in psoriasis [24]. Similarly, our study also demonstrated that the frequency of cTfh cells was higher in psoriasis patients than in healthy controls. Most importantly, we first observed that the number of Tfh cells was dramatically increased in psoriasis lesions compared with nonlesional skin tissues of psoriasis patients. However, although the number of infiltrated Tfh cells in lesions was increased in psoriasis, it was not correlated with PASI score in our study. This was possibly due to the limited sample size with only 10 psoriasis lesions collected in our study. We also investigated the CD19^+^ B cells in lesions by immunohistochemistry. Unfortunately, the results showed there were very few CD19^+^ B cells infiltrating in psoriasis lesions and no significant difference was found between lesional and nonlesional skin tissues of psoriasis patients (data not shown), and no ectopic GCs were observed in psoriasis lesions. Thus, additional psoriasis lesions need to be recruited to confirm whether Tfh cells participate in the pathogenesis through forming ectopic GCs in psoriasis lesions. Caruso et al. [25] have shown that IL-21 was highly expressed in the skin of individuals with psoriasis and stimulated human keratinocytes to proliferate. Higher levels of IL-17 and IFN-*γ* were also found in psoriasis lesions and correlated with disease severity [26, 27] Our results showed that cTfh cells were activated and secreted increased levels of IL-21, IL-17, and IFN-*γ* in peripheral blood of psoriasis (described below). We speculate that Tfh cells are also activated in lesions and secrete higher levels of inflammatory cytokines to participate in the development of psoriasis lesions. However, we need further investigation to confirm that Tfh cells are activated in psoriasis lesions. Wang et al. [28] have reported that the increased frequency of cTfh cells played vital roles in the pathogenesis of primary biliary cirrhosis (PBC). A higher frequency of cTfh cells was also found in patients with Henoch-Schönlein purpura [29]. These researches suggest that higher level of cTfh cells contributes to the activation of immune system. In addition, recent studies have reported that cTfh cells could be subdivided into several populations with different phenotypes, which had specialized role in immune system [30]. Different with previous study, Le Coz et al. [31] found that the frequency of cTfh cells showed no substantially difference between SLE patients and healthy individuals, but the subsets of cTfh cells were significantly different. Thus, our knowledge about cTfh cells is still in primary stage, and further studies need to clarify their phenotypic characteristics and functions in psoriasis.

Next, we investigate whether the function of cTfh cells is changed in psoriasis, which has not been studied in psoriasis. The molecular ICOS expression on Tfh cells is essential for Tfh cells generation. ICOS deficiency could induce a severe reduction of CXCR5^+^CD4^+^ T cells in germinal center [32]. In addition, ICOS-mediated signals could also promote Bcl-6 expression, a central regulator in Tfh cells development and function [10]. PD-1, another important receptor, acts as a key role in the activity of Tfh cells [16]. HLA-DR is a marker of T cell activation, and Ki-67 is a marker of T cell proliferation. Besides the surface receptors, cytokines secreted by Tfh cells are also important functional markers. IL-21 has been shown to be the key cytokine of Tfh cells, which can stimulate B cell proliferation and class switching [33]. Moreover, it has been shown that Tfh cells have the capacity to produce IL-17 and IFN-*γ* [34]. IL-17-producing Tfh cells have been shown to be involved in driving autoimmune responses [35]. Recent studies reported that the activated cTfh cells not only upregulated the expression of ICOS and IL-21 but also produce high level of IL-10 [36]. Thus, we detected the expression of ICOS, PD-1, HLA-DR, and Ki-67 on cTfh cells and secretion of IL-21, IL-17, IFN-*γ*, and IL-10 by cTfh cells in patients with psoriasis vulgaris. We found that the increasing levels of ICOS and PD-1 expression on cTfh cells had positive correlations with cTfh cell frequency, furthering the importance of ICOS and PD-1 for the generation of cTfh cells. Our data also showed that the expression of HLA-DR and Ki-67 on cTfh cells were significantly increased in psoriasis patients. The higher expression of HLA-DR indicated that cTfh cells were activated in psoriasis patients, and the increased level of Ki-67 suggested that cTfh cells were not quiescent cells in psoriasis patients. In addition, the levels of IL-21, IL-17, and IFN-*γ* secreted by cTfh cells were all statistically increased in psoriasis patients as well, which was further proved that cTfh cells were activated in psoriasis. Niu et al. [24] found the serum level of IL-21 was also increased in psoriasis and had positive correlations with cTfh frequency and disease severity. In PBC patients, the levels of IL-21, IL-17, and IFN-*γ* secreted by cTfh cells were all increased [28]. Jia et al. [37] represented that cTfh cell secreted lower levels of IL-21, IL-17, and IFN-*γ* in patients with hepatocellular carcinoma. Thus, either the activated or impaired function of cTfh cells contributes to the development of diseases. Taken together, we may speculate that the currently observed moleculesindicating the function of cTfh cells are closely related to the pathogenesis in psoriasis.

Previous study found that the increased frequency of cTfh cells was correlated with disease severity in psoriasis [24]. However, normal aging contributes to a decline in the function of the immune system at molecular level and cellular level [38]. The expression of numerous molecules required for Tfh cell differentiation and maintenance is dysregulated with age including ICOS, IL-21, and IL-12 [39]. Thus, if we want to determine whether cTfh cell frequency may be a biomarker of disease severity in psoriasis, we need to control the variables of age and disease duration. Our results showed that when the age or disease duration was considered as control variable, the percentage of cTfh cells was statistically correlated with disease severity in psoriasis. Furthermore, the levels of functional markers observed on cTfh cells except IL-10 have positive correlations with PASI score as well. Thus, our results confirmed that cTfh cell could be a biomarker to assess the disease severity.

To further confirm the role of cTfh cells in psoriasis and evaluate the corresponding therapeutic value, we observed these cells in psoriasis patients before and after treatment. Interestingly, after 1-month treatment with acitretin and a topical therapy, the frequency of cTfh cells was significantly decreased, accompanied with the decreased production of IL-21, IL-17, and IFN-*γ*. Previous studies demonstrated that acitretin could improve clinical symptoms of psoriasis through suppression of Th17 cells [40, 41]. Our data suggested that cTfh may also be a target of acitretin in psoriasis, thus possibly highlighted a potential therapeutic target in psoriasis.

## 5. Conclusion

In summary, this study first not only detects Tfh cells in psoriasis lesions and function of cTfh cells in psoriasis patients but also confirms the increased frequency and its positive correlation with disease severity in psoriasis patients. cTfh cells are activated in psoriasis, expressing high levels of ICOS, PD-1, HLA-DR, and Ki-67 and secreting increased levels of IL-21, IL-17, and IFN-*γ*. In addition, the frequency of cTfh cells and the production of cytokines are observed to be significantly decreased after 1-month treatment. Taken together, these findings indicate that activated cTfh cells contribute to the pathogenesis of psoriasis and may be a potential therapeutic target in psoriasis.

## Figures and Tables

**Table 1:** Clinical characteristics of participants.

Group	Patients with psoriasis vulgaris (PV)	Healthy controls (HC)	*P*
	*n* = 32	*n* = 13	
Age (years)	47.22 ± 10.35	42.47 ± 11.21	>0.05
Gender (F/M)	19/17	9/8	>0.05
Disease duration (year)	4.55 ± 2.97	—	
PASI scores (*n* = 32)	16.33 ± 8.06	—	—
Mild (*n* = 8)	7.17 ± 2.09	—	—
Moderate (*n* = 15)	14.80 ± 2.75	—	—
Severe (*n* = 9)	25.32 ± 6.61	—	—

**(a):** 

Control variables			cTfh cell frequency	PASI score	Age
-None-^a^	cTfh cell frequency	Correlation	1.000	.410	−.152
		Significance (2-tailed)	.	.020	.406
		df	0	30	30
	PASI score	Correlation	.410	1.000	.131
		Significance (2-tailed)	.020	.	.474
		df	30	0	30
	Age	Correlation	−.152	.131	1.000
		Significance (2-tailed)	.406	.474	.
		df	30	30	0

Age	cTfh cell frequency	Correlation	1.000	.439	
		Significance (2-tailed)	.	.014	
		df	0	29	
	PASI score	Correlation	.439	1.000	
		Significance (2-tailed)	.014	.	
		df	29	0	

^a^Cells contain zero-order (Pearson) correlations.

“.” means there is no correlation between cTfh cell frequency and cTfh cell frequency, PASI score and PASI score, and disease duration and disease duration. All the results are created by SPSS software.

**(b):** 

Control variables			cTfh cell frequency	PASI score	Disease duration
-None-^a^	cTfh cell frequency	Correlation	1.000	.410	.233
		Significance (2-tailed)	.	.020	.200
		df	0	30	30
	PASI score	Correlation	.410	1.000	.135
		Significance (2-tailed)	.020	.	.463
		df	30	0	30
	Disease duration	Correlation	.233	.135	1.000
		Significance (2-tailed)	.200	.463	.
		df	30	30	0

Disease duration	cTfh cell frequency	Correlation	1.000	.393	
		Significance (2-tailed)	.	.029	
		df	0	29	
	PASI score	Correlation	.393	1.000	
		Significance (2-tailed)	.029	.	
		df	29	0	

^a^Cells contain zero-order (Pearson) correlations.

“.” means there is no correlation between cTfh cell frequency and cTfh cell frequency, PASI score and PASI score, and disease duration and disease duration. All the results are created by SPSS software.
